# Immune Responses 6 Months After mRNA-1273 COVID-19 Vaccination and the Effect of a Third Vaccination in Patients with Inborn Errors of Immunity

**DOI:** 10.1007/s10875-023-01514-7

**Published:** 2023-05-26

**Authors:** Leanne P. M. van Leeuwen, Marloes Grobben, Corine H. GeurtsvanKessel, Pauline M. Ellerbroek, Godelieve J. de Bree, Judith Potjewijd, Abraham Rutgers, Hetty Jolink, Frank L. van de Veerdonk, Marit J. van Gils, Rory D. de Vries, Virgil A. S. H. Dalm, VACOPID Research Group, Eric C. M. van Gorp, Faye de Wilt, Susanne Bogers, Lennert Gommers, Daryl Geers, Marianne W. van der Ent, P. Martin van Hagen, Jelle W. van Haga, Bregtje A. Lemkes, Annelou van der Veen, Rogier W. Sanders, Karlijn van der Straten, Judith A. Burger, Jacqueline van Rijswijk, Khadija Tejjani, Joey H. Bouhuijs, Karina de Leeuw, Annick A. J. M. van de Ven, S. F. J. de Kruijf-Bazen, Pieter van Paassen, Lotte Wieten, Petra H. Verbeek-Menken, Annelies van Wengen, Anke H. W. Bruns, Helen L. Leavis, Stefan Nierkens

**Affiliations:** 1 Department of Viroscience, Erasmus MC University Medical Center Rotterdam, Rotterdam, The Netherlands; 2 Travel Clinic, Erasmus MC University Medical Center Rotterdam, Rotterdam, The Netherlands; 3 Department of Medical Microbiology and Infection Prevention, Amsterdam Institute for Infection and Immunity, Amsterdam UMC, University of Amsterdam, Amsterdam, The Netherlands; 4 Department of Internal Medicine, UMC Utrecht, Utrecht, The Netherlands; 5 Department of Infectious Diseases, Amsterdam UMC, Amsterdam, The Netherlands; 6 Department of Internal Medicine, Division Nephrology and Clinical Immunology, Maastricht UMC, Maastricht, The Netherlands; 7 Department of Rheumatology and Clinical Immunology, UMC Groningen, Groningen, The Netherlands; 8 Department of Infectious Diseases, Leiden University Medical Center, Leiden, The Netherlands; 9 Department of Internal Medicine, Radboud University Medical Center, Nijmegen, The Netherlands; 10 Department of Internal Medicine, Division of Allergy & Clinical Immunology, Erasmus MC University Medical Center Rotterdam, Doctor Molewaterplein 40, 3015 GD Rotterdam, The Netherlands; 11 Department of Immunology, Erasmus MC University Medical Center Rotterdam, Doctor Molewaterplein 40, 3015 GD Rotterdam, The Netherlands

**Keywords:** Inborn errors of immunity, primary immunodeficiency disorders, SARS-CoV-2, mRNA-1273 COVID-19 vaccine, immunogenicity, antibody response, T cell response

## Abstract

**Purpose:**

Patients with inborn errors of immunity (IEI) are at increased risk of severe coronavirus disease-2019 (COVID-19). Effective long-term protection against COVID-19 is therefore of great importance in these patients, but little is known about the decay of the immune response after primary vaccination. We studied the immune responses 6 months after two mRNA-1273 COVID-19 vaccines in 473 IEI patients and subsequently the response to a third mRNA COVID-19 vaccine in 50 patients with common variable immunodeficiency (CVID).

**Methods:**

In a prospective multicenter study, 473 IEI patients (including X-linked agammaglobulinemia (XLA) (*N* = 18), combined immunodeficiency (CID) (*N* = 22), CVID (*N* = 203), isolated or undefined antibody deficiencies (*N* = 204), and phagocyte defects (*N* = 16)), and 179 controls were included and followed up to 6 months after two doses of the mRNA-1273 COVID-19 vaccine. Additionally, samples were collected from 50 CVID patients who received a third vaccine 6 months after primary vaccination through the national vaccination program. SARS-CoV-2-specific IgG titers, neutralizing antibodies, and T cell responses were assessed.

**Results:**

At 6 months after vaccination, the geometric mean antibody titers (GMT) declined in both IEI patients and healthy controls, when compared to GMT 28 days after vaccination. The trajectory of this decline did not differ between controls and most IEI cohorts; however, antibody titers in CID, CVID, and isolated antibody deficiency patients more often dropped to below the responder cut-off compared to controls. Specific T cell responses were still detectable in 77% of controls and 68% of IEI patients at 6 months post vaccination. A third mRNA vaccine resulted in an antibody response in only two out of 30 CVID patients that did not seroconvert after two mRNA vaccines.

**Conclusion:**

A similar decline in IgG titers and T cell responses was observed in patients with IEI when compared to healthy controls 6 months after mRNA-1273 COVID-19 vaccination. The limited beneficial benefit of a third mRNA COVID-19 vaccine in previous non-responder CVID patients implicates that other protective strategies are needed for these vulnerable patients.

**Supplementary Information:**

The online version contains supplementary material available at 10.1007/s10875-023-01514-7.

## Introduction

Inborn errors of immunity (IEI), also referred to as primary immunodeficiencies (PIDs), are a heterogeneous group of inborn disorders affecting a single or multiple component(s) of the immune system. This results in an increased susceptibility to infections, autoimmune complications, autoinflammatory diseases, allergies and malignancies. Absent or disturbed responses to vaccination are characteristic of various IEIs, which hamper adequate protection against vaccine-preventable diseases. Coronavirus disease-2019 (COVID-19)-associated morbidity and mortality is reported to be higher in unvaccinated patients with IEI [1, 2]. Sustained presence of immune responses targeting the causative agent, severe acute respiratory syndrome coronavirus-2 (SARS-CoV-2), are of great importance for these patients, especially in the face of emerging SARS-CoV-2 variants.

We recently assessed immunogenicity, tolerability and safety of the mRNA-1273 COVID-19 vaccine (Moderna) in over 500 patients with various IEI. Except for patients with X-linked agammaglobulinemia (XLA), the majority of IEI patients seroconverted 28 days following the second vaccination, but Spike (S)-specific IgG titers were significantly lower in patients with common variable immunodeficiency (CVID), combined B and T cell immunodeficiency (CID), isolated antibody deficiencies (IgG subclass deficiency ± IgA deficiency, specific polysaccharide antibody deficiency (SPAD)) and undefined antibody deficiencies when compared to controls. A number of CVID patients, especially those with (multiple) non-infectious complications, did not mount detectable antibody responses at all. Despite the absent antibody response in XLA patients, these patients had comparable T cell responses to controls, whereas in patients with CVID significantly lower T cell responses were found [3]. Similar findings were reported in other studies that assessed the immunogenicity of COVID-19 vaccines in IEI patients [4–8].

In the current phase of the pandemic, it is important to obtain insight in long-term vaccine immunogenicity, and the effect of additional COVID-19 vaccinations in patients with IEI. In healthy individuals, a rapid antibody response after the primary COVID-19 vaccination regimen was observed, but also substantial waning over time [9–12]. More rapid decay of antibodies was reported in elderly and patients using immune suppressive medication [13]. A recent study demonstrated that antibody levels were significantly lower 6 months after second vaccination in IEI patients when compared to antibody levels at 1 month after second vacation [14]. Direct comparisons between IEI patients and controls without IEI has to our knowledge not yet been performed 6 months after second vaccination. Additionally, the magnitude and longevity of the T cell response in IEI patients compared to controls remains unclear.

In this study, we investigated SARS-CoV-2-specific antibodies, neutralizing antibodies, and T cell responses 6 months after the second vaccination with the mRNA-1273 COVID-19 vaccine in 473 IEI patients compared to 179 controls. Patients were stratified into cohorts of patients with XLA, CVID, CID, isolated antibody deficiencies (IgG subclass deficiency ± IgA deficiency, SPAD), phagocyte defects and undefined antibody deficiencies. Additionally, we assessed whether a third mRNA COVID-19 vaccine induced seroconversion in CVID patients who were seronegative after two vaccinations, and whether the magnitude of the antibody and T cell response increased after a third vaccine in CVID patients who were seropositive after two vaccinations.

## Methods

### Ethical Statement

The Vaccination Against COvid in Primary Immune Deficiencies (VACOPID) study is a prospective, controlled, multicenter study performed among IEI patients from seven university hospitals in the Netherlands. The study adheres to the principles of the Declaration of Helsinki and was approved by the Dutch Central Committee on Research Involving Human Subjects (CCMO, NL7647.078.21, EudraCT number 2021–000,515-24), the Medical Research Ethics Committee from Erasmus University Medical Center (MEC-2021–0050) and the local review boards of all other participating centers. All participants provided written informed consent before enrollment.

### Study Design and Participants

Detailed information about the study design and inclusion and exclusion criteria was previously described [3]. All study participants received two mRNA-1273 COVID-19 vaccinations with an interval of 28 days according to the Dutch COVID-19 vaccination program. The first vaccinations were administered between March 9, 2021 and April 15, 2021. Blood samples for this study were collected at the following time points: 28 days (May–June 2021) and 6 months after second vaccination (October–November 2021). Results of the immunogenicity of the mRNA-1273 COVID-19 vaccine 28 days after second vaccination have been described previously [3]. An amendment to the original study protocol was submitted and approved by the Medical Research Ethics Committee from Erasmus University Medical Center to investigate the immunogenicity of a third mRNA COVID-19 vaccination in our patient cohort. The Dutch vaccination program recommended a third mRNA-based COVID-19 vaccine to patients with CID, CVID patients with immunosuppressant use, or in specific cases when a medical specialist had reasonable arguments to make an exception to the aforementioned indications, based on proven or assumed non-response [15, 16]. These vaccines were administered from October 2021 onwards. Within our study cohort, we collected blood samples from 50 CVID patients 4–8 weeks after they received a third mRNA-based COVID-19 vaccine from public health services.

In total, blood samples were collected from 473 patients with IEI. These patients were stratified into different cohorts (Table 1). The number of participants per center was proportional to the total number of IEI patients treated in each of the centers. The number of participants per cohort was also proportionally distributed across all centers. The clinically comparable cohorts isolated IgG subclass deficiency ± IgA deficiency and SPAD were analyzed as one group. Additionally, blood samples from 179 controls, defined as not diagnosed with IEI, were collected. These controls were relatives of the IEI patients, such as partner or other household- or family members. Baseline characteristics, including medical history and medication use, were recorded. Participants who contracted COVID-19 before start of the study were not included in the main analysis but analyzed separately. The definition of an infection with SARS CoV-2 was either a history of a positive PCR and/or an N-specific IgG titer above 42.2 BAU/ml.

**Table 1:** Baseline characteristics

	Controls *N* = 163	X-linked agammaglo-bulinemia (XLA)^a^ *N* = 17	Combined Immunode-ficiency (CID) *N* = 20	Common Variable Immunode-ficiency (CVID) *N* = 188	Isolated IgG subclass deficiency ± IgA deficiency (*N* = 108) and specific polysaccharide antibody deficiency (SPAD) (*N* = 53) *N* = 161	Phagocyte defects *N* = 15	Undefined antibody deficiency^b^ *N* = 16	Other^c^ *N* = 8
Male, n (%)	93 (57.1)	16 (94.1)	5 (25)	75 (39.9)	50 (31.1)	12 (80.0)	8 (50.0)	4 (50.0)
P-value	Ref	.003^A^	.001^A^	.001^B^	< .001^B^	.106^A^	.607^A^	-
Median age (IQR)	54 (42–62)	42 (36–52)	38 (29–54)	48 (34–60)	54 (45–63)	41 (33–44)	51 (46–58)	52 (44–62)
P-value	Ref	.013^C^	.001^C^	.005^C^	.458^C^	.001^C^	.568^C^	-
Immunoglobulin replacement therapy, n (%)	0	17 (100)	8 (40)	170 (94)	87 (54.0)	1 (6.7)	9 (56.3)	3 (37.5)
IEI with ≥ 1 non-infectious complication, n (%)	N/A	4 (23.5)	12 (60)	109 (58.0)	41 (25.5)	9 (6.0)	6 (37.5)	-
Immunosuppressive medication used in past 2 years and during the study, n (%)	4 (2.5)	3 (17.6)	7 (35)	58 (39)	27 (16.7)	1 (6.7)	0	-
Prednisone / other corticosteroid treatment	3	2	3	44	23	1		
Azathioprine				9	2			
Anti-TNF-a			1	7	2			
Hydroxychloroquine			2	3	3	1		
Mycophenolate mofetil				6	1			
Other DMARDs		1		5	1			
Methotrexate	1			4	2			
Anti-CD20^d^				6				
Calcineurin inhibitors			1	3	1			
Anti-Il6				2				
JAK inhibitors			1	1				

^A^Fisher exact test. ^B^Pearson’s Chi-Squared test, ^C^Wilcoxon rank-sum test. ^a^In the text this cohort is referred to as XLA, although it also includes one participant with autosomal dominant agammaglobulinemia (TCF3 mutation). ^b^Patients with primary hypogammaglobulinaemia and intact cellular immunity who do not fulfill diagnostic criteria of any of the other primary antibody deficiencies. ^c^Patients with an unknown classification of their IEI, high B cells numbers or hyper IgM syndrome. ^d^Also includes anti-CD20 therapies used before 2 years prior to the start of the study. Ref: reference. TNF: tumor necrosis factor. DMARD: disease modifying anti-rheumatic drugs

### Measurement of Humoral and Cellular Immune Responses

The assays to evaluate humoral and cellular immune responses were described previously [3]. Briefly, spike (S)-specific IgG and receptor binding domain (RBD) protein-specific IgG were measured by a quantitative Luminex assay and expressed as international Binding Antibody Units per mL (BAU/ml) [17, 18]. Participants with S-specific IgG above 44.8 BAU/ml were considered seropositive [19]. Nucleocapsid (N)-specific IgG antibodies were measured via Luminex assay to identify participants who contracted COVID-19 before the start of the study or after the second or third vaccination. Cut-off values were previously calculated using pre-pandemic samples (*n* = 113) and samples of PCR-confirmed SARS-CoV-2 adults (*n* = 282) [18, 19]. However, for N-specific IgG, we calculated a new cut-off value for which 100% of previously measured healthy donor sera were negative, resulting in a sensitivity of 87% and specificity of 100% [18]. N-specific antibody titers above 42.2 BAU/ml were considered positive. All cut-off values used and their sensitivity and specificity can be found in online resource 1. We made a selection of samples to be tested for the presence of neutralizing antibodies, based on the observed variation in S-specific IgG titers. Due to a large variation in S-specific IgG titers all CID (*n* = 19) and CVID patients (*n* = 180) were selected for neutralization assays. We selected participants from the IgG/SPAD cohort (*n* = 36) and controls (*n* = 23) of one participating center (Erasmus MC) because of a smaller variation in the observed S-specific IgG titers. SARS-CoV-2 neutralization was determined using a pseudovirus system based on SARS-CoV-2 S and HIV-1-NL43 ΔEnv-NanoLuc reporter virus and HEK293T-ACE2 cells and 50% inhibitory serum dilutions (ID_50_) are expressed as IU/ml [20, 21]. All IgG and neutralizing antibody titers were normalized to the WHO International Standard for anti-SARS-CoV-2 immunoglobulin (NIBSC 20/136). SARS-CoV-2-specific T cell responses were measured in samples obtained from four out of seven study sites with two different Interferon-gamma (IFN-ɣ) release assays (IGRA, QIAGEN or EuroImmun). No IGRAs were performed in the other participating centers due to limited worldwide availability of the assays at the start of the study. The QIAGEN assay was performed on samples from the university hospitals of Leiden and Rotterdam, while the EuroImmun assay was used in the university hospitals of Utrecht and Nijmegen. IGRA results were expressed in IU/ml after subtraction of the negative control value [22, 23].

### Statistical Analysis

Baseline characteristics were described for IEI patients and controls. Categorical variables are displayed as numbers and percentages and analyzed with Pearson’s Chi-Squared test or Fisher’s exact test, depending on the number of observations. Continuous variables are presented as mean ± standard deviation (SD) or as median ± interquartile range (IQR), depending on data distribution. Fold changes are displayed as the mean of individual fold changes. Results of the immunological assays were displayed in figures and text with geometric means ± 95% confidence intervals (CI). Continuous variables were analyzed using the Wilcoxon rank-sum test. Paired data (differences within cohorts over time) were analyzed using Wilcoxon singed rank test. Antibody decay was calculated using an exponential decay formula, where the slope was calculated as log10(slope) = (log10(S-specific IgG titer at 6 months)-log10(S-specific IgG titer at 28 days)/difference in time between these measurements (in days). According to this model, IgG titers at 6 months could be calculated as follows: IgG titer at 28 days after second vaccination time * slope raised to the power of the time difference in days. *P*-values below 0.05 were considered statistically significant. Bonferroni correction was used in case of multiple testing. Spearman’s ρ test was used to perform correlation analysis. Our sample size calculation has been published previously [3].

### Software

Study data were collected in an online electronic data capture system (Castor©, Amsterdam, the Netherlands), compliant to the General Data Protection Regulation (GDPR). R studio was used for statistical analyses. Graphs were drafted with GraphPad PRISM, version 9.1.2 (San Diego, CA, USA).

## Results

### Baseline Characteristics

**Fig. 1:**
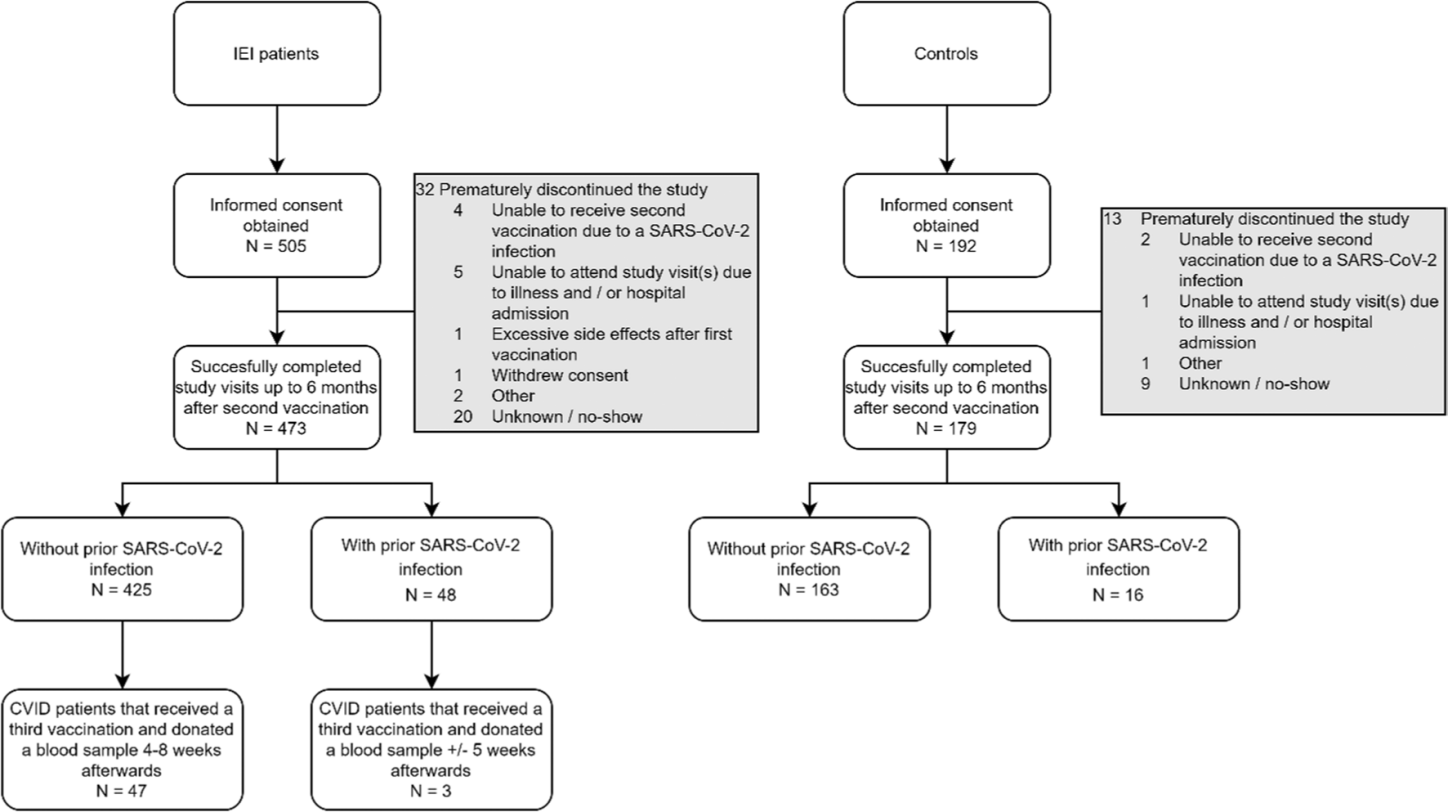
Subject enrollment and outcome after four visits. In total, 697 participants signed informed consent (505 IEI patients, 192 controls). Forty-five participants did not complete the four visits (32 IEI patients, 13 controls). The 652 remaining participants (473 IEI patients, 179 controls) were included and described in detail in Table 1. Sixty-four participants (48 IEI patients, 16 controls) were considered as COVID-19 recovered patients. Fifty CVID patients received a third vaccination and donated a blood sample afterwards

Blood samples were collected from 652 participants (473 with IEI and 179 controls) at 28 days and 6 months after second vaccination (Fig. 1). Out of the 652 study participants, 64 (48 IEI patients and 16 controls) had evidence of a SARS CoV-2 infection until 6 months after second vaccination. Four hundred twenty-five IEI patients and 163 controls had not contracted a SARS-CoV-2 infection until 6 months after second vaccination and were eligible for further analysis.

Baseline characteristics of the patient and control groups are summarized in Table 1. The percentage of males differed significantly between the control cohort and the XLA-, CID-, CVID-, and IgG/SPAD cohort (*P* values in Table 1). Median ages of the patient cohorts were lower compared to the median age of the control cohort, except for the IgG/SPAD cohort and the undefined antibody deficiency cohort which were similar to the controls (Table 1). The mean interval between timing of the second vaccination and the evaluation at 6 months thereafter was 184 days (SD 9.3). Blood samples after a third vaccination were collected from 50 patients (Fig. 1). The mean interval between the timing of the second vaccination and the third vaccination was 198 days (SD 20 days). This third vaccination was administered with a mean interval of 16 days after the 6-month study visit. The mean interval between the third vaccination and blood sampling was 35 days (SD 10 days). Four IEI patients received a third vaccine dose before samples were obtained at 6 months and were excluded for this part of the analyses.

### S-Specific Antibodies Decline over Time at Similar Rates for Controls and IEI Patients

**Fig. 2:**
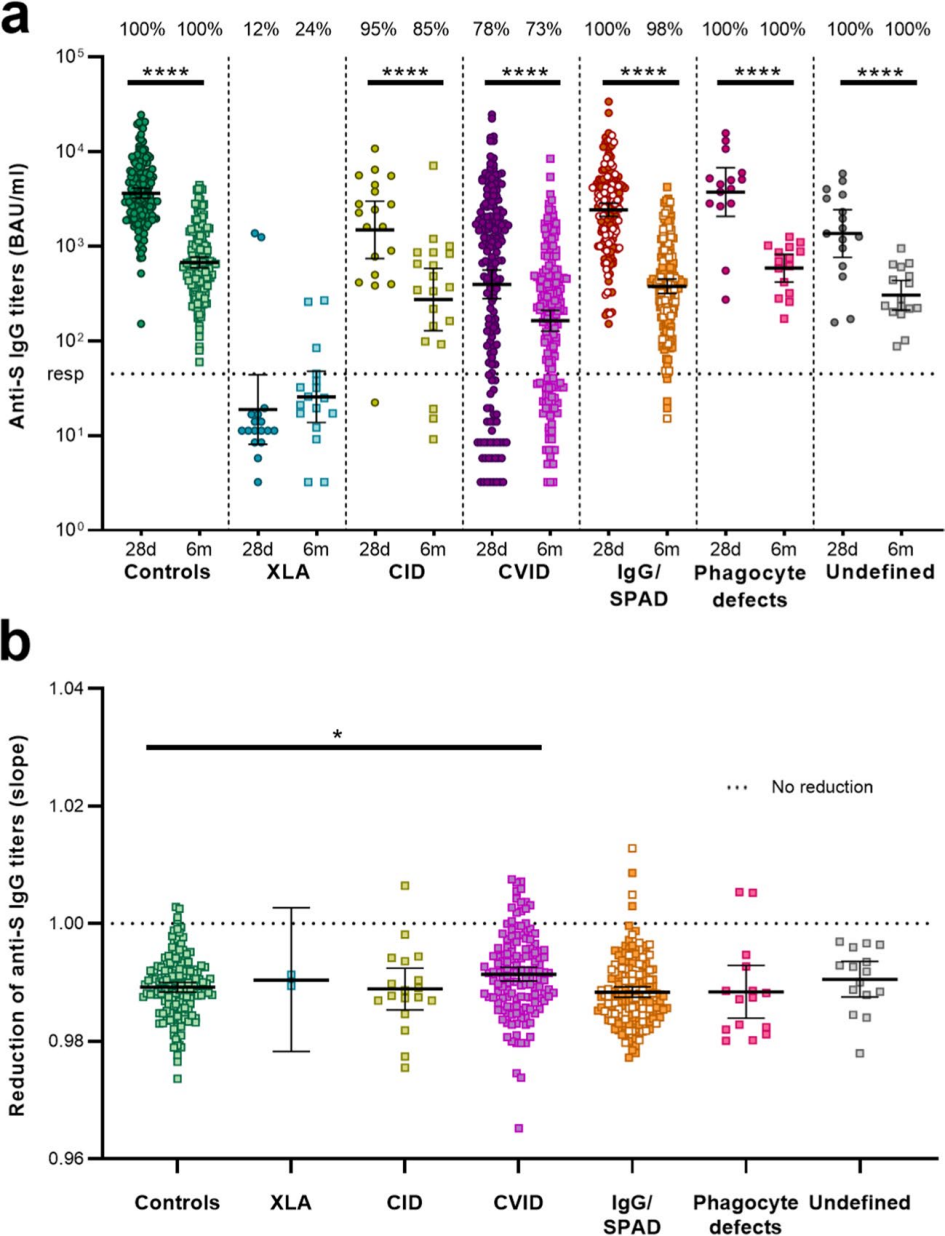
SARS-CoV-2-specific IgG and decay between 28 days and 6 months after second vaccination. (**a**) S-specific IgG measured by Luminex for controls and different cohorts of IEI patients 28 days and 6 months after the second vaccination. Results at 28 days after the second vaccination were published previously. Results are expressed in binding antibody units per milliliter (BAU/mL). The dotted line is the pre-defined responder cut-off (resp). The percentage of responders is indicated above the graph. Line indicates the geometric mean, error bars indicate the 95% confidence interval. IgG titers at 28 days and 6 months were compared per cohort using the Wilcoxon paired signed rank test. (**b**) Decay of S-specific IgG expressed as the slope between the two time points determined by an exponential decay model. Only participants with a response 28 days after the second vaccination were included for this analysis. A slope of 1.00 represents no decay in S-specific IgG between the two time points. Slopes between controls and IEI patients were compared using a Wilcoxon rank-sum test with correction for multiple comparisons. In both graphs, the SPAD cohort is indicated with white symbols while the IgG cohort is indicated with orange symbols. S = Spike, XLA = X-linked agammaglobulinemia, CID = Combined Immunodeficiency, CVID = Common Variable Immunodeficiency, IgG = Isolated IgG subclass deficiency ± IgA deficiency, SPAD = Specific polysaccharide antibody deficiency, Undefined = Undefined antibody deficiency, * = *P* < .05, **** = *P* < .0001

To determine the decay of SARS-CoV-2 S-specific antibody titers, these were evaluated in sera obtained 6 months after second vaccination (Fig. 2A, Online Resource 2). SARS-CoV-2 S-specific IgG titers at 28 days after second vaccination were previously determined [3]. The GMT of S-specific IgG in the control cohort declined 7.7-fold from 3633 BAU/ml (95% CI [3213–4110]) 28 days after second vaccination to 673 BAU/ml (95% CI [590–768]) 6 months after second vaccination (*P* < 0.001) (Fig. 2A) (Online Resource 3). S-specific IgG in patients with CID, CVID, IgG, SPAD, phagocyte defects, and undefined antibody deficiencies significantly declined over the course of 6 months as well (9.1-fold, 5.9-fold, 8.7-fold, 11.2-fold, and 6.4-fold, respectively; *P* < 0.001) (Fig. 2A). Seropositivity rates in the control group, in patients with phagocyte defects or with an undefined antibody deficiency remained 100% 6 months after the second vaccination. Seropositivity rates were 85% in the CID cohort, 73% in the CVID cohort, and 98% in the IgG/SPAD cohort 6 months after second vaccination (compared to 95%, 78%, and 100% after 28 days, respectively). Low seropositivity rates were found in the XLA cohort after 6 months, which was comparable to 28 days after second vaccination (24% and 12%, respectively). S-specific IgG titers strongly correlated with RBD-specific IgG titers (Spearman* r* = 0.938, *p* < 0.0001, Online Resource 4).

Using an exponential model, we calculated the rate of decay (slope) of S-specific IgG between 28 days after the second vaccination and 6 months later, in order to determine whether the decay is different between IEI patients and controls (Fig. 2B). Non-responders 28 days after the second vaccination were excluded from this analysis. Decay of S-specific IgG titers was comparable between controls and IEI cohorts, except for the CVID cohort. The mean antibody response in CVID patients 28 days after second vaccination was lower than in the other cohorts but declined slower compared to controls (*P* = 0.003). A potential complication in this analysis is that 295 IEI patients (69.4%) were on regular immunoglobulin replacement therapy (IGRT). These preparations are collected from healthy plasma donors, and it was shown that SARS-CoV2 specific antibody levels in these preparations increase over time. Therefore, we evaluated the effect of IGRT on antibody levels in the CID-, CVID-, and IgG/SPAD cohorts. We did not find less decay of SARS-CoV-2 IgG levels in patients who receive IGRT compared to patients who did not receive IGRT in these cohorts (see Online Resource 5), indicating that receiving IGRT did not influence total SARS-CoV-2-specific antibody titers.

Participants who had a SARS-CoV-2 infection before the first vaccination (15 controls, 11 CVID and 18 IgG/SPAD patients) were analyzed separately; samples from other patient groups were excluded due to the small sample size. IGRT was used by 91% of these CVID- and 61% of the IgG/SPAD patients, which was comparable with the rates of the non-infected cohorts (*p* = 1 and *p* = 0.6251 respectively). S-specific IgG titers declined significantly at 6 months for participants with prior COVID-19 compared to 28 days after second vaccination (*P* = 0.0040 for controls, *P* = 0.0039 for CVID and *P* < 0.001 for IgG/SPAD) (Online Resource 6). However, S-specific IgG titers 6 months after second vaccination were higher for prior infected participants compared to participants without a prior infection, significant for the controls (*P* = 0.005) and IgG/SPAD cohort (*P* < 0.001), but not for the CVID cohort (*P* = 0.395). The decay of antibodies in participants with a prior SARS-CoV-2 infection was similar to participants without a prior SARS-CoV-2 infection in both the control cohort as well as the CVID and IgG/SPAD cohort (Online Resource 6). One control, four CVID, and five IgG/SPAD patients were infected with SARS-CoV-2 between their second vaccination and the 6 months thereafter. Six of them were diagnosed by PCR testing, and four had N-specific IgG titers above 42.2 BAU/ml. S-specific IgG antibodies above responder cut-off were present in nine of these participants 28 days after second vaccination. The remaining participant missed the study visit 28 days after second vaccination due to the SARS-CoV-2 infection.

### Neutralizing Antibodies Decline Between 28 Days and 6 Months After the Second Vaccination

**Fig. 3:**
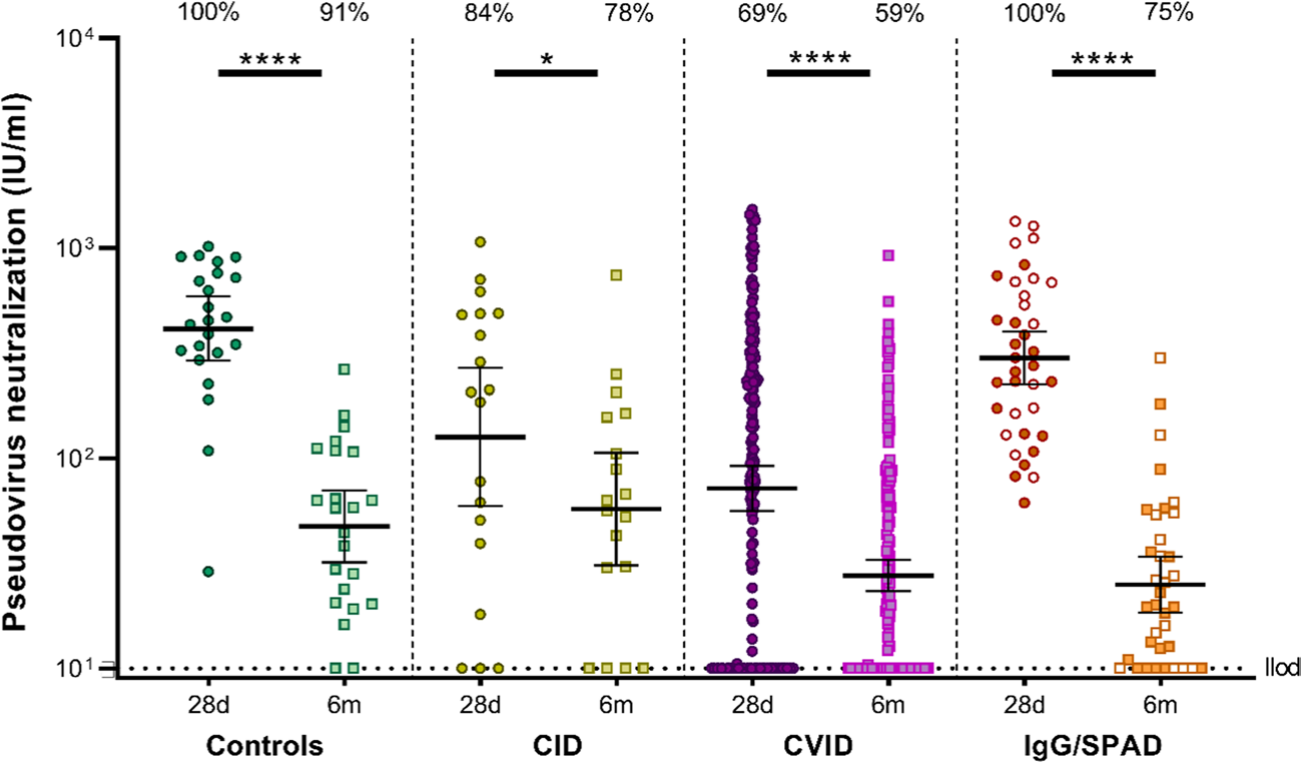
SARS-CoV-2 neutralizing antibodies 28 days and 6 months after the second COVID-19 vaccination. Neutralizing antibody titers as 50% inhibitory dilution (ID_50_) determined by SARS-CoV-2 pseudovirus neutralization assay 28 days and 6 months after the second COVID-19 vaccination expressed as international units/milliliter (IU/mL). Line indicates the geometric mean, error bars indicate the 95% confidence interval. The lower limit of detection (llod) of the pseudovirus neutralization assay is 10 IU/ml. The percentage of participants with detectable neutralizing antibodies is indicated above the graph. Neutralizing antibodies were compared between the two time points using the Wilcoxon paired signed rank test. CID = Combined Immunodeficiency, CVID = Common Variable Immunodeficiency, IgG = Isolated IgG subclass deficiency ± IgA deficiency, SPAD = Specific polysaccharide antibody deficiency, * = *P* < .05, **** = *P* < .0001

Based on the observed differences in S-specific IgG titers, a selection of samples was analyzed for the presence of neutralizing antibodies in a pseudovirus neutralization test with the ancestral SARS-CoV-2: in 19 CID patients, 180 CVID patients, 36 IgG/SPAD patients, and 23 controls. Neutralizing antibodies were detected in all controls 28 days after the second vaccination, but the responder rate declined to 91% at 6 months after second vaccination (Online Resource 7). As for IEI patients, neutralizing activity against the ancestral SARS-CoV-2 was detected in 78% of the CID patients, 59% of the CVID patients and 75% of the IgG/SPAD patients 6 months after the second vaccination (compared to 84%, 69%, and 100% 28 days after second vaccination). Quantitatively, neutralizing titers also decreased significantly in all cohorts (Fig. 3) (Online Resource 7). Neutralizing titers correlated significantly with S-specific IgG at both time points (*r* = 0.852 at 28 days and *r* = 0.797 at 6 months, both *P* < 0.0001) (Online Resource 8).

### T Cell Responses Decline Between 28 Days and 6 Months After the Second Vaccination

**Fig. 4:**
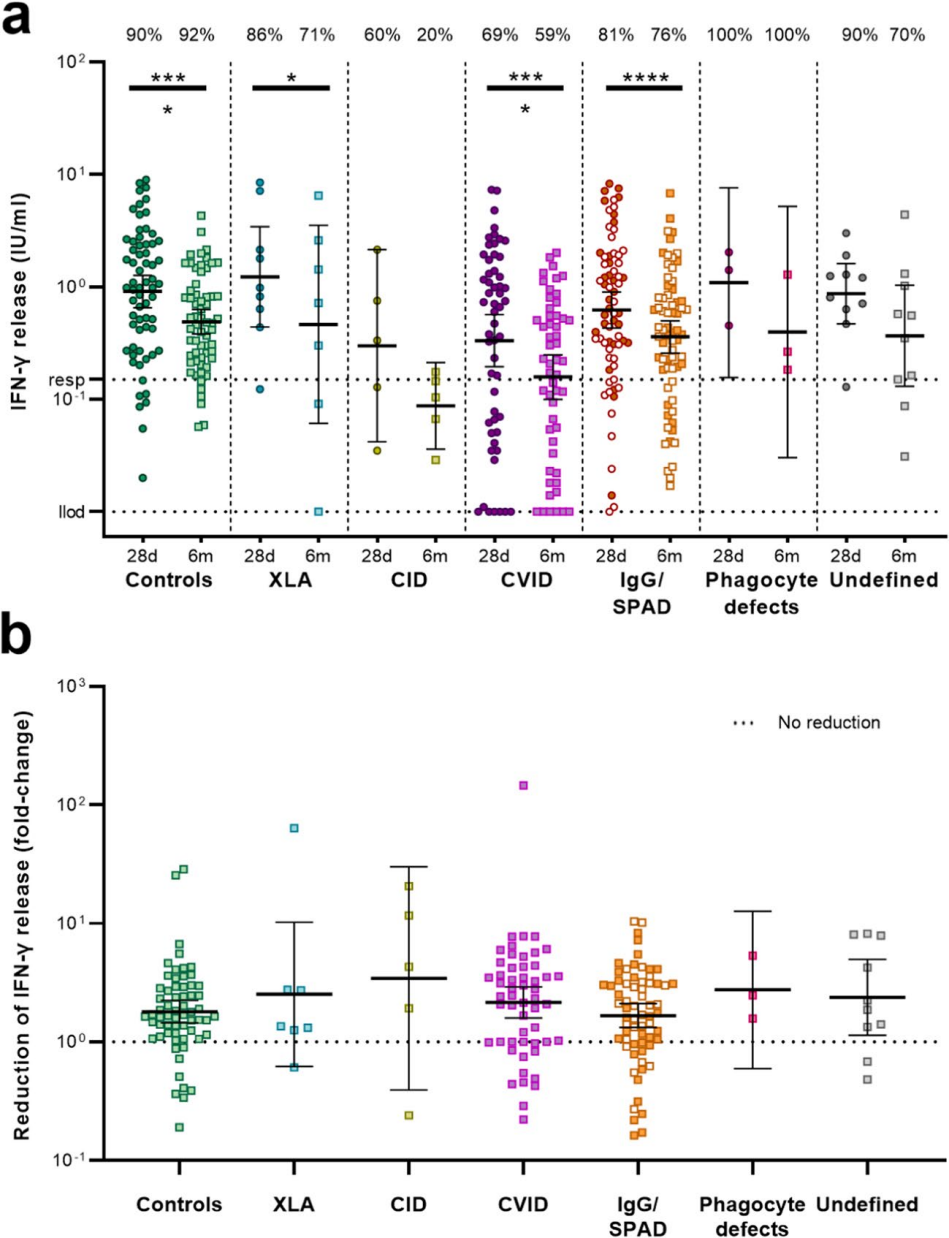
SARS-CoV-2-specific T cell responses 28 days and 6 months after the second COVID-19 vaccination. (**a**) SARS-CoV-2-specific T cell responses measured by an IFN-γ release assay (QIAGEN) after stimulation of whole blood 28 days and 6 months after the second vaccination. Lower limit of detection (llod) is .01 IU/ml and responder cut off (resp) was .15 IU/ml. The percentage of responders is indicated above the graph. Results are expressed as international units/milliliter (IU/mL). Line indicates the geometric mean, error bars indicate the 95% confidence interval. Within each cohort, IFN-γ levels at 28 days and 6 months were compared using Wilcoxon paired signed rank test. (**b**) Fold change IFN-γ levels between the two timepoints. A fold-change larger than 1 indicates that SARS-CoV-2-specific T-cells responses were lower at 6 months compared to 28 days. Fold changes were compared between the controls and IEI patients using a Wilcoxon rank-sum test with correction for multiple comparisons. In both graphs, the SPAD cohort is indicated with white symbols while the IgG cohort is indicated with orange symbols. XLA = X-linked agammaglobulinemia, CID = Combined Immunodeficiency, CVID = Common Variable Immunodeficiency, IgG = Isolated IgG subclass deficiency ± IgA deficiency, SPAD = Specific polysaccharide antibody deficiency, Undefined = Undefined antibody deficiency, * = *P* < .05, **** = *P* < .0001

SARS-CoV-2-specific T cell responses were measured in samples obtained from four study sites using the QIAGEN- (Fig. 4) or EuroImmun assay (Online Resource 9)). In the QIAGEN assay, using a peptide pool covering the S protein (Ag2), an IFN-γ response was still detectable (cut-off of IFN-γ levels > 0.15 IU/ml) 6 months after second vaccination in 71% (5/7) participants of the XLA cohort, 20% (1/5) of the CID cohort, 59% (30/51) of the CVID cohort, 76% (51/67) of the IgG/SPAD cohort, 100% of the phagocyte defects cohort (3/3), and 70% (7/10) of the undefined antibody deficiency cohort. For controls, specific T cell responses were detected in 90% (56/62, 28 days) and 92% (56/61, 6 months). IFN-γ levels significantly declined in the controls (*P* < 0.0001), XLA (*P* = 0.047), CVID (*P* < 0.0001) and IgG/SPAD (*P* < 0.0001) cohorts 6 months after the second vaccination compared to 28 days after vaccination (Fig. 4A), but not in the CID (*P* = 0.19), phagocyte defects (*P* = 0.25) and undefined antibody deficiency cohorts (*P* = 0.23). As a measure for waning of T cell responses, we calculated the fold change in IFN-γ levels of the QIAGEN assay between 28 days and 6 months after the second vaccination, and compared these between IEI patients and controls (Fig. 4B). None of the IEI cohorts showed a larger reduction of IFN-γ levels compared to the controls. The majority of the participants with both a low IgG titer (< 44.8 BAU/ml) and a low IFN-γ levels 6 months after second vaccination (< 0.15 IU/ml) were CVID patients (Online Resource 10).

### Third SARS CoV-2 Vaccination in a Subset of Patients with IEI

**Fig. 5:**
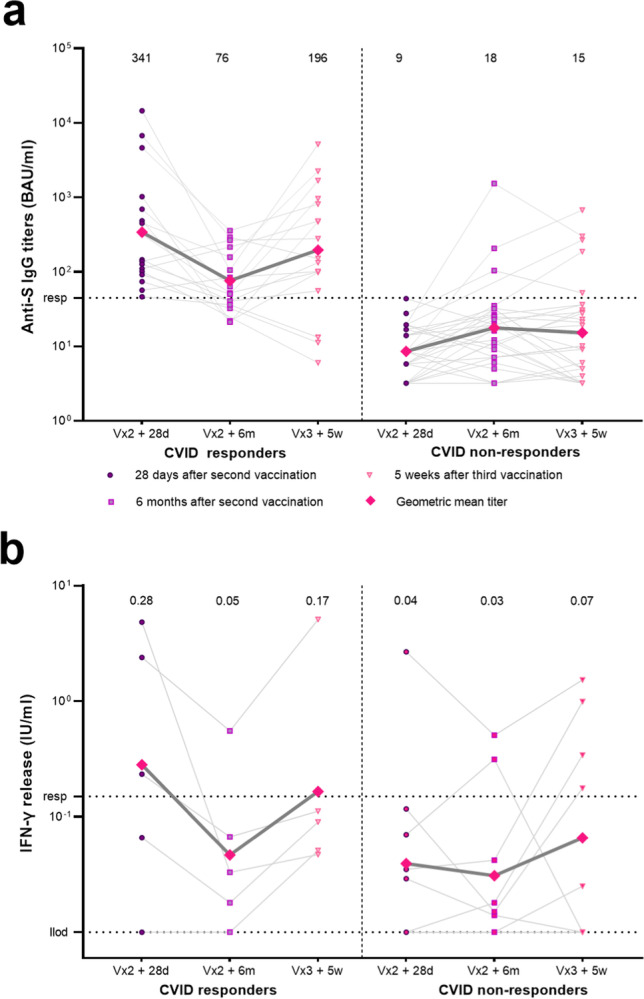
SARS-CoV-2 -specific IgG titers and T cell responses 28 days and 6 months after the second COVID-19 vaccination, and 5 weeks after the third vaccination. (**a**) S-specific IgG titers measured by Luminex is for CVID patients classified as responder (left) or non-responders (right) based on antibody titers 28 days after second vaccination. Displayed timepoints are 28 days after second vaccination (dots), 6 months after the second vaccination (squares) and 5 weeks after third vaccination (triangles). Results are expressed in binding antibody units per milliliter (BAU/mL). The diamond symbols indicate the geometric mean titers, which are also specified above the data points. The dotted line is the responder cut-off (44.8 BAU/ml). (**b**) SARS-CoV-2-specific T cell responses measured by the QIAGEN interferon-gamma release assay. Lower limit of detection is .01 IU/ml. The dotted line is the pre-defined responder cut-off (.15 IU/ml). Results are expressed as international units/milliliter (IU/mL). The diamond symbols indicate the geometric mean titer. S = spike, CVID = Common Variable Immunodeficiency, ** = *P* < .01

We measured immune responses in 50 IEI patients who received an additional vaccination through the national vaccination program. Three patients had a history of COVID-19 before or during the study and were excluded from this analysis. The remaining 47 participants were all CVID patients. Forty-one patients received the BNT162b2 vaccine as third vaccination, three received the mRNA-1273 vaccine and for three patients the vaccine type was unknown (Table 2). In 17 of these CVID patients we had detected low IgG responses 28 days after the second vaccination. Third vaccination increased the GMT significantly in these 17 low-responders (geometric mean of 76 BAU/ml 6 months after second vaccination to 196 BAU/ml after third vaccination *P* = 0.008) (Fig. 5). Thirty CVID patients included in this sub-study were classified as non-responders 28 days after second vaccination. In our previous study, we had already found that nonresponding CVID patients had more noninfectious complications and used more immunosuppressive medication compared to responding CVID patients [3]. In this sub-study, a majority (63%) of the non-responders had a history of multiple auto-inflammatory- or immunoproliferative complications (Table 2). In three non-responders, an IgG response was present 6 months after second vaccination. These patients were either slow-responders and/or should have had a SARS-CoV-2 infection that was not detected by N-specific antibodies. Only two out of the 27 remaining seronegative CVID patients did develop a serological response after the third vaccination (Fig. 5). Theoretically, an increase in S-specific antibodies could be explained by IGRT which all non-responders received. However, S-specific antibody levels were still estimated to be low in IGRT preparations administered at the time of the third vaccination, which is confirmed by the minimal increase in antibody levels of the remaining non-responders. Nevertheless, the administration of a preparation with above-average concentrations for that period cannot completely be ruled out. Immunosuppressant medications were used equally in the non-responder vs the responder group (Table 2). More non-responders used multiple types of immunosuppressive medication compared to responders (53% vs 30%) although this was not statistically significant. SARS-CoV-2-specific T cell responses after third vaccination were measured in samples obtained from two study sites with the QIAGEN Interferon-gamma release assays (*N* = 14). Geometric mean IFN-γ levels increased after third vaccination, although most levels were still below the responder cut-off (Fig. 5B). In 5 of the 14 CVID patients in which both antibody- and T cell measurements were performed after third vaccination, both responses were absent.

**Table 2:** Clinical characteristics of CVID patients receiving a third vaccination

	Non-responder* after first 2 vaccinations *N* = 30	Responder* after first 2 vaccinations *N* = 17	*P* value
Male, *n* (%)	14 (47%)	8 (47%)	1^A^
Median age (min, max)	51 (30–71)	51 (27–71)	.527^B^
Non-infectious complications present, *n* (%)	25 (83%)	15 (88%)	1^A^
No complication	5 (17%)	2 (12%)	1^A^
1 complication	6 (20%)	10 (59%)	.011^A^
> 1 complication	19 (63%)	5 (29%)	.036^A^
Autoimmune cytopenia	10 (33%)	1 (6%)	-
Other autoimmune diseases	10 (33%)	10 (59%)	-
Enteropathy	3 (10%)	2 (12%)	-
Malignancy	5 (17%)	2 (12%)	-
Lymphoproliferative diseases	16 (53%)	2 (12%)	-
GLILD	12 (40%)	2 (12%)	-
Other granulomatous diseases	3 (10%)	1 (6%)	-
Immunosuppressive medication used in past 2 years and during the study, *n* (%)	19 (63%)	10 (59%)	.766^A^
Multiple types of immunosuppressive medication used in past 2 years and during the study, *n* (%)	10 (53%)	3 (30%)	.434^A^
Prednisone / other corticosteroid treatment	14	5	
Azathioprine	2	2	
Anti-TNF-a	2	3	
Hydroxychloroquine	1	1	
Mycophenolate mofetil	2	2	
Other DMARDs	2	1	
Methotrexate	2	1	
Calcineurin inhibitors	2	0	
Anti-CD20 (year of treatment)	4 (2014, 2017, 2019, 2020)	2 (2017, 2021)	
Anti-IL-6	2	0	
JAK inhibitor	1	0	
IGRT (%)	30 (100%)	16 (94%)	.362^A^
Third vaccination type	Pfizer 26 Moderna 2 Unknown 2	Pfizer 15 Moderna 1 Unknown 1	-

*The responder cut-off was defined as S-specific IgG antibodies > 44.8 BAU/ml. ^A^: Fisher exact test. ^B^: Wilcoxon rank-sum test U test. ^¥^Also includes anti-CD20 therapies used before 2 years prior to the start of the study. *S* spike, *GLILD* granulomatous-lymphocytic interstitial lung disease, *TNF* tumor necrosis factor, *DMARD* disease-modifying anti-rheumatic drugs, *IGRT* immunoglobulin replacement therapy

## Discussion

This study investigated the longevity of antibody and T cell responses 6 months after two mRNA-1273 COVID-19 vaccines in a large group of IEI patients. Binding and neutralizing antibodies significantly declined 6 months after the second vaccination both in IEI patients and controls, but we found no evidence for a faster decline in IEI patients. In addition, we did not observe antibody responses after a third mRNA-1273 COVID-19 vaccine in previous CVID non-responders, while we did observe a serological boosting in CVID patients that previously responded to vaccination.

A decay of antibody levels upon completion of a primary COVID-19 vaccination regimen between 28 days and 6 months after the second vaccination has been reported in the general population [24]. In the current study, we observed that the decline in binding antibodies up to 6 months after second vaccination was comparable between controls and IEI patients, indicating that antibody levels in IEI patients do not undergo a faster decay. However, especially in patients with lower antibody responses at 28 days after vaccination [3], antibody levels can drop below the responder cut-off after 6 months in these cohorts, which was observed for the CID-, CVID-, and IgG/SPAD cohorts. This resulted in a higher proportion of controls still having measurable neutralizing antibody titers against SARS-CoV-2 after 6 months when compared to patients with IEI. Similar findings were observed in other immunocompromised patients, for example kidney transplant recipients or dialysis patients [25]. We also observed similar decline patterns for neutralizing antibody titers, which are regarded as a correlate of protection against symptomatic SARS-CoV-2 infection [26].

Coronavirus-specific T cell responses are durable, and in contrast to antibodies retain reactivity to emerging antigenically distinct SARS-CoV-2 variants [12, 27–29]. Here, we observed similar decline of T cell responses between controls and IEI patients. As expected, (most) XLA patients had no detectable antibodies, but still showed SARS-CoV-2-specific T cell responses that were comparable to controls. These T cell responses remained detectable up to 6 months after the second vaccination in the majority of XLA patients. The level of protection mediated exclusively by T cells against severe disease upon breakthrough infections in these patients is unclear. Further studies in XLA patients are warranted to elucidate the potential role of T cells as a correlate of protection against severe COVID-19. In two XLA patients, S-specific antibodies were detectable both at 28 days and at 6 months after second vaccination. Since anti-S antibodies were absent or low in IGRT batches administered at the time of blood collections (March-November 2021), this could be explained by the fact that hypogammaglobinemia is described in a minority of XLA patients [30–32].

The clinical phenotypes of CVID are diverse, explaining the varying immunogenicity of mRNA-based COVID-19 vaccines in this patient group [3, 4, 33]. Although the decay of antibodies in CVID patients who did respond after second vaccination appeared slightly slower, this is most likely due to limitations in assay sensitivity below the responder cut-off. Furthermore, a slight increase in antibody levels was observed in CVID patients with absent or very low antibody responses after the second vaccination and in the XLA cohort (Online Resource 1). These results could be affected by the fact that these patients are often treated with immunoglobulin preparations (IGRT), in which levels of SARS-CoV-2 antibodies are increasing [31]. However, the assessment of SARS-CoV-2-specific antibody concentrations in consecutive batches of commercially available immunoglobulins until January 2022 demonstrated that antibodies are not consistently detected [31]. In addition, we did not find a reduced decay of IgG levels in patients who did receive IGRT compared to patients who did not receive IGRT.

Partially as a consequence of declining antibody titers, and the reduced reactivity of antibodies with emerging SARS-CoV-2 variants, booster vaccinations were implemented for the general population [12, 28, 34]. Large observational studies confirmed that booster vaccinations increased protection against severe COVID-19 in the general population compared to 2-dose regimens [35, 36]. To better protect vulnerable patient groups, a third vaccination was added to the primary vaccination regimen for certain immunocompromised patients in particular in the Netherlands. As a consequence, a number of patients in the CVID cohort received a third vaccination. In patients that did not mount a detectably antibody response shortly after the second vaccination, this third vaccination did not result in an increase of SARS-CoV-2-specific antibodies in the majority of patients. This seems consistent with recently published studies [14, 37]. In addition, the more impaired response to vaccinations of CVID patients with multiple complications was also observed in other studies [33, 38]. Although we observed an increase in T cell responses after the third vaccination in a small subset of patients, T cell responses were still undetectable in more than half of the cases. It is therefore crucial to consider other measures of protection from severe COVID-19 for CVID patients with auto-immunity or immune dysregulation that seem not to mount an adequate immune response after repeated vaccinations. These strategies could include the (prophylactic) use of SARS-CoV-2 specific monoclonal antibodies, or therapeutic administration of antiviral drugs [31, 37, 39].

This study has several limitations. First, our cohort includes only individuals initially vaccinated with the mRNA-1273 COVID-19 vaccine. A recent study reported on 6 months immunogenicity after BNT162b2- vaccination in a cohort of predominantly CVID patients with seropositivity rates of 55.6% [14]. These rates are lower than the rates we found in our CVID cohort. However, due to the use of different antibody measurement methods and clinical variability in the CVID diagnosis, these results may not be comparable. Second, we did not have enough statistical power to study the effect of different types of immune suppressive medication. Third, the exponential decay model is only a rough approximation of the actual decay curve. This model was mainly used in our study to compare between cohorts, as has been done in other studies [25]. Fourth, the number of CVID patients that received a third vaccination was relatively low and limited T cell data were available of these CVID patients.

In conclusion, we showed in a large group of IEI patients that these have lower antibody levels compared to controls 6 months after second vaccination. However, the decay of antibodies in IEI patients was comparable to that in controls. Repeated vaccination of prior non-responders did not prove effective, in contrast to what has been observed in kidney transplant recipients [40]. In these patients, other protective measures should be considered. Future studies should address the effect of repeated booster vaccinations in IEI patients compared to controls, combined with in-depth immunological assessments to gain more insight in the immune responses after COVID-19 vaccination in patients with IEI. These insights will be beneficial to current- and future vaccination strategies against COVID-19, but also against other pathogens.

## Supplementary Information

Below is the link to the electronic supplementary material.

Supplementary file1 (DOCX 1198 KB)

## Data Availability

The datasets analyzed during the current study are available from the corresponding author on reasonable request.
