# Supplementary material for: Immune Responses 6 Months After mRNA-1273 COVID-19 Vaccination and the Effect of a Third Vaccination in Patients with Inborn Errors of Immunity
Source: J Clin Immunol. 2023 May 26;43(6):1104–17. doi: 10.1007/s10875-023-01514-7 (PMC10212732; doi:10.1007/s10875-023-01514-7)
Supplement: Supplementary file 1 — Supplementary file1 (DOCX 1198 KB) [file 10875_2023_1514_MOESM1_ESM.docx]

**Supplementary information to:**

Immune responses six months after mRNA-1273 COVID-19 vaccination and the effect of a third vaccination in patients with inborn errors of immunity

Leanne P.M. van Leeuwen^1,2*^, MD, Marloes Grobben^3*^, MSc, Corine H. GeurtsvanKessel^1⸙†^, MD PhD, Pauline M. Ellerbroek^4⸙†^, MD PhD, Godelieve J. de Bree^5⸙†^, MD PhD, Judith Potjewijd^6⸙†^, MD, Abraham Rutgers^7⸙†^, MD PhD, Hetty Jolink^8⸙†^, MD PhD, Frank L. van de Veerdonk^9⸙†^, MD PhD, Marit J. van Gils^3ǂ†^, PhD, Rory D. de Vries^1ǂ†^, PhD, Virgil A.S.H. Dalm^10,11ǂ†^, MD PhD, VACOPID research group

^1^ Department of Viroscience, Erasmus MC University Medical Center Rotterdam, The Netherlands.

^2^ Travel Clinic, Erasmus MC University Medical Center Rotterdam, The Netherlands

^3^ Department of Medical Microbiology and Infection Prevention, Amsterdam Institute for Infection and Immunity, Amsterdam UMC, University of Amsterdam, Amsterdam, The Netherlands

^4^ Department of Internal Medicine, UMC Utrecht, The Netherlands

^5^ Department of Infectious Diseases, Amsterdam UMC, The Netherlands

^6^ Department of Internal Medicine, Division Nephrology and Clinical Immunology, Maastricht UMC, The Netherlands

^7^ Department of Rheumatology and Clinical Immunology, UMC Groningen, The Netherlands

^8^ Department of Infectious Diseases, Leiden University Medical Center, The Netherlands

^9^ Department of Internal Medicine, Radboud university medical center Nijmegen, The Netherlands

^10^ Department of Internal Medicine, Division of Allergy & Clinical Immunology, Erasmus MC University Medical Center Rotterdam, The Netherlands.

^11^ Department of Immunology, Erasmus MC University Medical Center Rotterdam, The Netherlands

^*^The first two authors equally contributed to this manuscript, ^⸙^ these authors equally contributed as second author.

^ǂ^ These authors equally contributed to this work as shared last authors. ^†^ On behalf of the VACOPID Study Consortium.

*Corresponding author:*

Virgil A.S.H. Dalm

Erasmus University Medical Center Rotterdam

Doctor Molewaterplein 40, 3015 GD

Rotterdam, The Netherlands

[v.dalm@erasmusmc.nl](about:blank)

This appendix has been provided by the authors to give readers additional information about the work

**Table of content:**

Online Resource 1: Supplementary table 1 3

*Sensitivity and Specificity of SARS-CoV-2 antibodies measured by Luminex assay*

Online Resource 2: Supplementary fig. 1 4 *SARS-CoV-2 S-specific IgG decay per participant*

Online Resource 3: Supplementary table 2 5 *Geometric mean titers 28 days and six months after second vaccination*

Online Resource 4: Supplementary fig. 2 6

*RBD-specific IgG at 28 days and six months after second vaccination and correlation between S- and RBD-specific IgG*

Online Resource 5: Supplementary fig. 3 7

*The decay of S-specific IgG titers between 28 days and six months after second vaccination in patients with- and without immunoglobulin replacement therapy*

Online Resource 6: Supplementary fig. 4 8

*SARS-CoV-2 S-specific IgG and decay between 28 days and six months after second vaccination for participants with a prior SARS-CoV-2 infection*

Online Resource 7: Supplementary table 3 9 *Neutralizing antibodies titers and responder rates 28 days and six months after second vaccination*

Online Resource 8: Supplementary fig. 5 10

*Correlation between neutralizing antibodies and -specific binding antibodies*

Online Resource 9: Supplementary fig. 6 11

*SARS-CoV-2-specific T-cell responses 28 days and six months after the second COVID-19 vaccination obtained with a EuroImmun assay*

Online Resource 10: Supplementary fig. 7 12

*Correlation between IgG titers and T-cell responses six months after second COVID- 19 vaccination*

**Online Resource 1: Supplementary table 1**

**Sensitivity and Specificity of Luminex assays for SARS-CoV-2-specific antibodies**

|  | **Cut-off** | **Sensitivity** | **Specificity** |
| --- | --- | --- | --- |
| Spike-protein specific IgG | 44,8 BAU/mL | 97% | 96% |
| Receptor binding domain (RBD) protein specific IgG | 22,0 BAU/mL | 96% | 91% |
| Nucleocapsid specific IgG | 42,2 BAU/mL | 100% | 87% |

**Online Resource 2: Supplementary fig. 1**

| 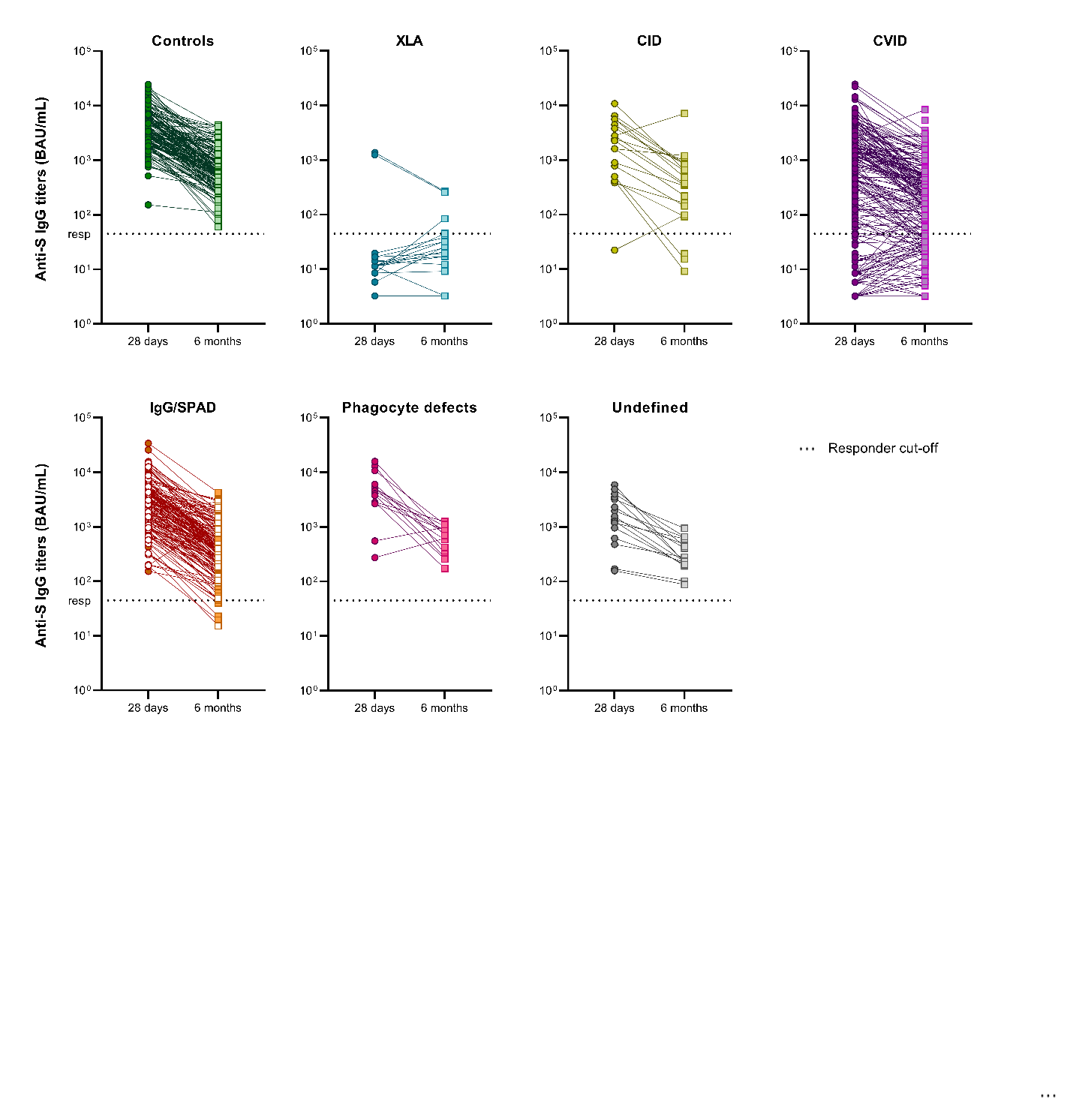 |
| --- |
| **Fig. 1 SARS-CoV-2 S-specific IgG decay per participant**. S-specific IgG decay measured by Luminex per individual participant. Results are expressed in binding antibody units per milliliter (BAU/mL). The dotted line is the responder cut-off. S = Spike, XLA = X-linked agammaglobulinemia, CID = Combined Immunodeficiency, CVID = Common Variable Immunodeficiency, IgG = Isolated IgG subclass deficiency ± IgA deficiency, SPAD = Specific polysaccharide antibody deficiency, Undefined = Undefined antibody deficiency. |

**Online Resource 3: Supplementary table 2**

**Geometric mean titers 28 days and six months after second vaccination**

|  | **N** | **GMT + 28 days**  **[95% CI]** | **GMT + six months**  **[95% CI]** | **P-value** | **Fold change** |
| --- | --- | --- | --- | --- | --- |
| Controls | 163 | 3634 [3213-4110] | 673 [590-768] | <.001 | 7.7 |
| X-linked agammaglobulinemia(XLA) | 17 | 19 [8-44] | 26 [14-48] | 0.14 | 1.3 |
| Combined Immunodeficiency (CID) | 20 | 1492 [743-2997] | 273 [128-585] | <.001 | 9.1 |
| Common Variable Immunodeficiency (CVID) | 185 | 395 [278-561] | 163 [127-210] | <.001 | 5.9 |
| Isolated IgG subclass deficiency ± IgA deficiency (IgG) / Specific polysaccharide antibody deficiency (SPAD) | 160 | 2425 [2064-2848] | 378 [318-449] | <.001 | 8.7 |
| Phagocyte defects | 15 | 3739 [2064-6775] | 585 [418-819] | <.001 | 11.2 |
| Undefined | 15 | 1362 [761-2438] | 304 [212-435] | <.001 | 6.4 |

IgG titers at 28 days and six months were compared per cohort using the Wilcoxon paired signed rank test.

**Online Resource 4: Supplementary fig. 2**

| 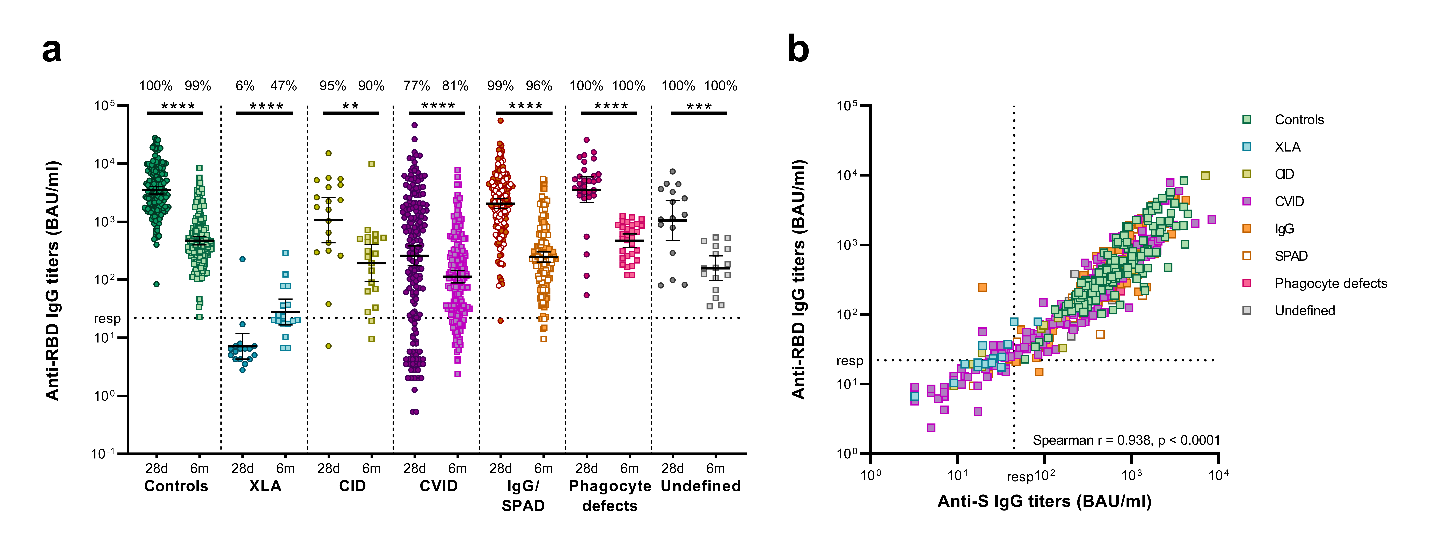 |
| --- |
| **Fig. 2 RBD-specific IgG at 28 days and six months after second vaccination and correlation between S- and RBD-specific IgG**  (a) RBD-specific IgG measured by Luminex for controls and different cohorts of IEI patients 28 days and six months after the second vaccination. Results at 28 days after the second vaccination were published previously. Results are expressed in binding antibody units per milliliter (BAU/mL). The dotted line is the pre-defined responder cut-off (22.0 BAU/mL)(resp). The percentage of responders is indicated above the graph. Line indicates the geometric mean, error bars indicate the 95% confidence interval. IgG titers at 28 days and six months were compared per cohort using the Wilcoxon paired signed rank test. (b) Correlation between RBD-specific IgG and S-specific IgG six months after second COVID-19 vaccination (right panel). The dotted vertical line is the responder cut-off for S-specific IgG (44.8 BAU/ml). The dotted horizontal line is the responder cut-off for RBD S-specific IgG (22.0 BAU/ml). Spearman’s ρ test was used to perform correlation analysis. XLA = X-linked agammaglobulinemia, CID = Combined Immunodeficiency, CVID = Common Variable Immunodeficiency, IgG = Isolated IgG subclass deficiency ± IgA deficiency, SPAD = Specific polysaccharide antibody deficiency, Undefined = Undefined antibody deficiency. ** = P<0.01, *** = P<.001 **** = P<.0001. |

**Online Resource 5: Supplementary fig. 3**

| 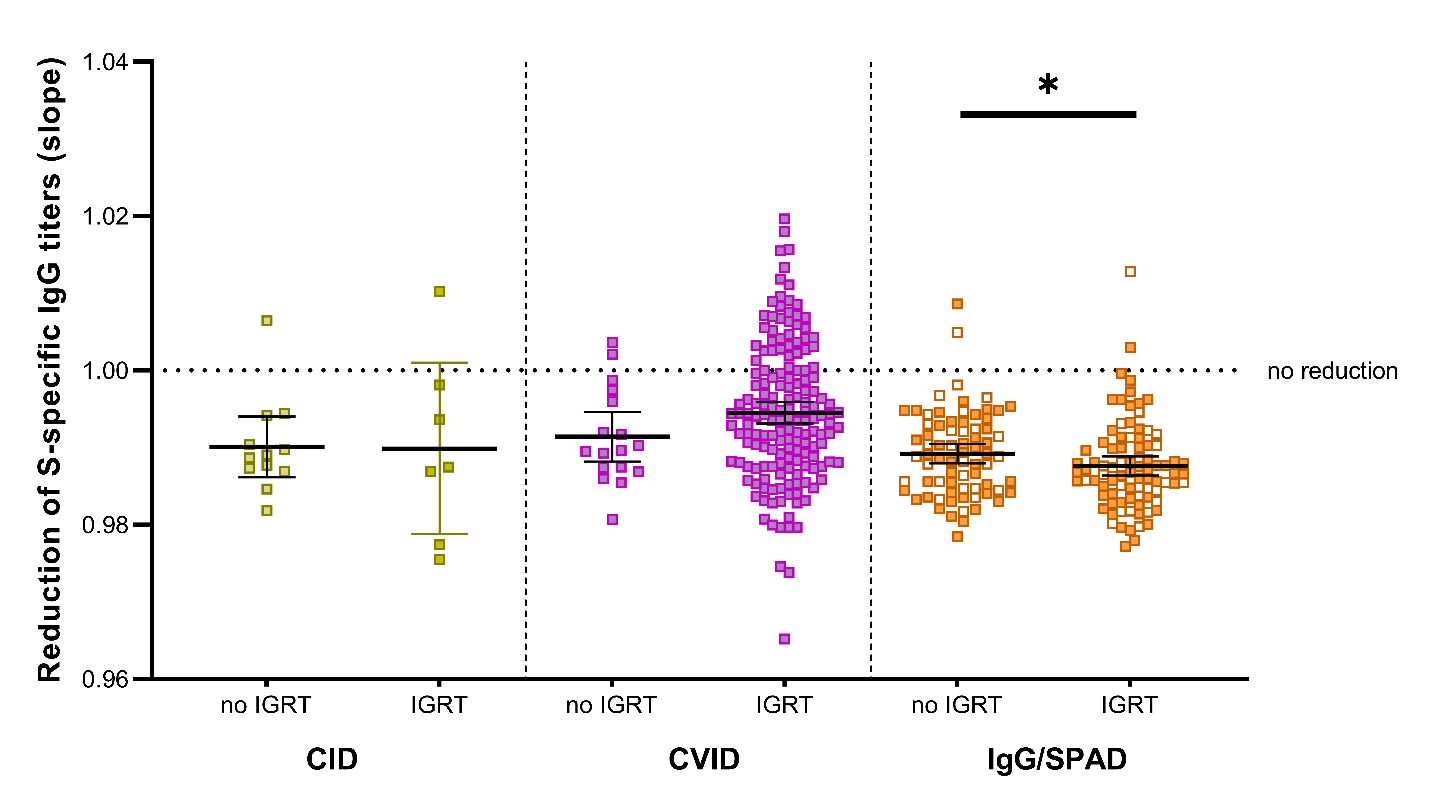 |
| --- |
| **Fig. 3 The decay of S-specific IgG titers between 28 days and six months after second vaccination in patients with- and without immunoglobulin replacement therapy**. Decay of S-specific IgG expressed as the slope between the time points 28 days and 6 months after the second vaccination, determined by an exponential decay model. A slope of 1.00 represents no decay in S-specific IgG between the two time points. Slopes between IEI patients with and without IGRT treatment were compared using a Wilcoxon rank-sum test. The SPAD cohort is indicated with white symbols while the IgG cohort is indicated with orange symbols. S = Spike, CID = Combined Immunodeficiency, CVID = Common Variable Immunodeficiency, IgG = Isolated IgG subclass deficiency ± IgA deficiency, SPAD = Specific polysaccharide antibody deficiency, IGRT = immunoglobulin replacement therapy, * = P<.05. |

**Online Resource 6: Supplementary fig. 4**

| 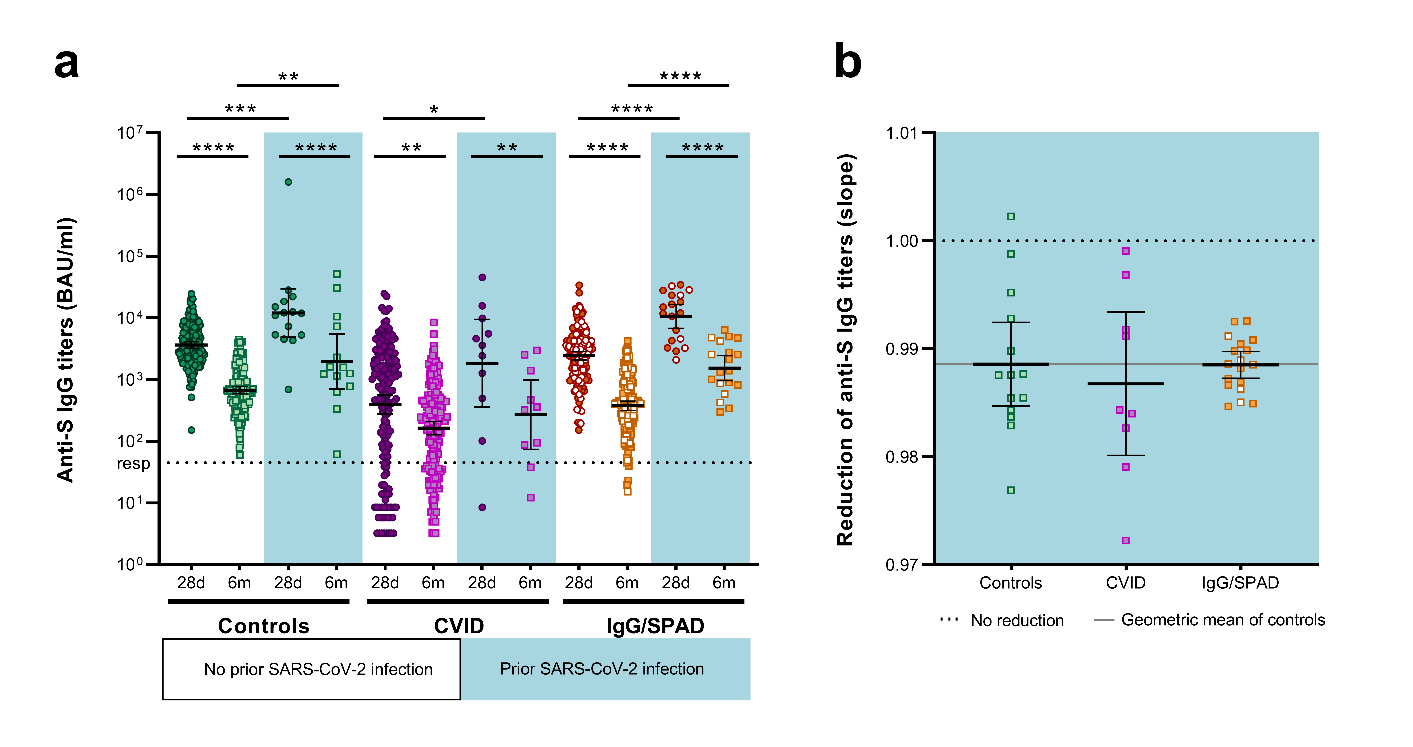 |
| --- |
| **Fig. 4** **SARS-CoV-2 S-specific IgG and decay between 28 days and six months after second vaccination for participants with a prior SARS-CoV-2 infection (before first vaccination).** (a) S-specific IgG measured by custom Luminex assay for participants with (white background) and without (blue background) a prior SARS-CoV-2 infection from the controls, the CVID and the SPAD cohort at 28 days after the second SARS-CoV-2 vaccination and at six months after the second vaccination. Results are expressed in binding antibody units per milliliter (BAU/mL). The line is the geometric mean and the error bars the 95% confidence interval. The dotted line is the pre-defined responder cut-off (resp). IgG titers at 28 days and six months were compared per cohort and IgG titers between prior infected and not prior infected participants were compared at both timepoints using the Wilcoxon paired signed rank test. (b) The decay of S-specific IgG titers in previously SARS-CoV-2 infected participants expressed as the slope between the two timepoints determined by an exponential decay model. Only participants with a response 28 days after the second vaccination were included. A slope of 1.00 represents no decay in S-specific antibodies between the two time points. Slopes between the controls and each group of IEI patients were compared using a Wilcoxon rank-sum test with correction for multiple comparisons. In both graphs, the SPAD cohort is indicated with white symbols while the IgG cohort is indicated with orange symbols. S = Spike, CVID = Common Variable Immunodeficiency, IgG = Isolated IgG subclass deficiency ± IgA deficiency, SPAD = Specific polysaccharide antibody deficiency, * = P<0.05, ** = P<0.01, *** = P<.001 **** = P<.0001 |

**Online Resource 7: Supplementary table 3**

**Neutralizing antibodies titers and responder rates 28 days and six months after second vaccination**

|  | **28 days** | | **six months** | |  |
| --- | --- | --- | --- | --- | --- |
| **Cohort** | **>llod (%)** | **GMT [95% CI]** | **>llod (%)** | **GTM [95% CI]** | **P-value^A^ (GMT)** |
| Controls | 23/23 (100%) | 412 [290-587] | 19/21 (91%) | 48 [32-70] | <.0001 |
| CID | 16/19 (84%) | 126 [59-269] | 14/18 (78%) | 57 [24-106] | .010 |
| CVID | 124/180 (69%) | 72 [56-92] | 103/176 (59%) | 28 [24-33] | <.0001 |
| IgG/SPAD | 36/36 (100%) | 299 [224-401] | 27/36 (75%) | 25 [18-34] | <.0001 |

1. Wilcoxon signed rank test. Llod: lower limit of detection. GMT: geometric mean titer.

**Online Resource 8: Supplementary fig. 5**

| 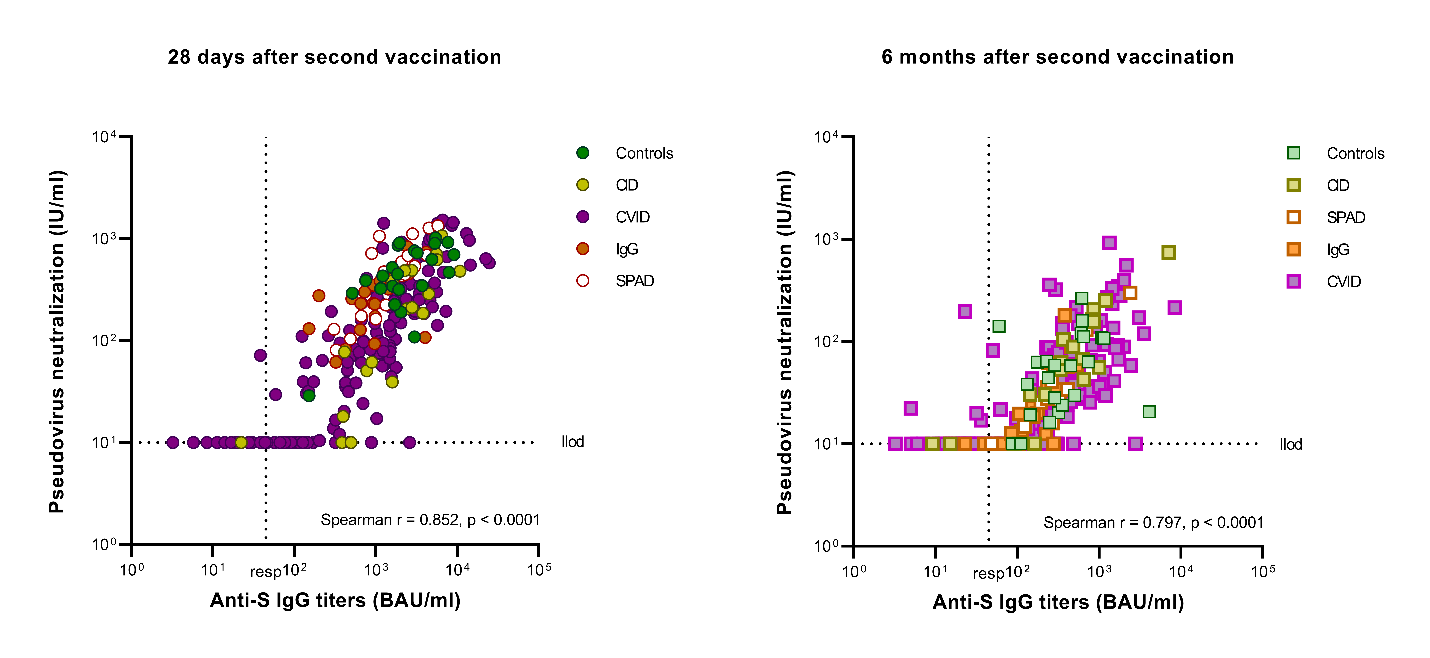 |
| --- |
| **Fig. 5 Correlation between neutralizing antibodies and S-specific IgG**. Correlation between neutralizing antibodies and IgG 28 days (left panel) and six months after second COVID-19 vaccination (right panel). The lower limit of detection of the pseudovirus neutralization assay is 10 IU/ml. The dotted vertical line is the responder cut-off of the Luminex assay (44.8 BAU/ml). Spearman’s ρ test was used to perform correlation analysis. XLA = X-linked agammaglobulinemia, CID = Combined Immunodeficiency, CVID = Common Variable Immunodeficiency, IgG = Isolated IgG subclass deficiency ± IgA deficiency, SPAD = Specific polysaccharide antibody deficiency, Undefined = Undefined antibody deficiency. |

**Online Resource 9: Supplementary fig. 6**

| 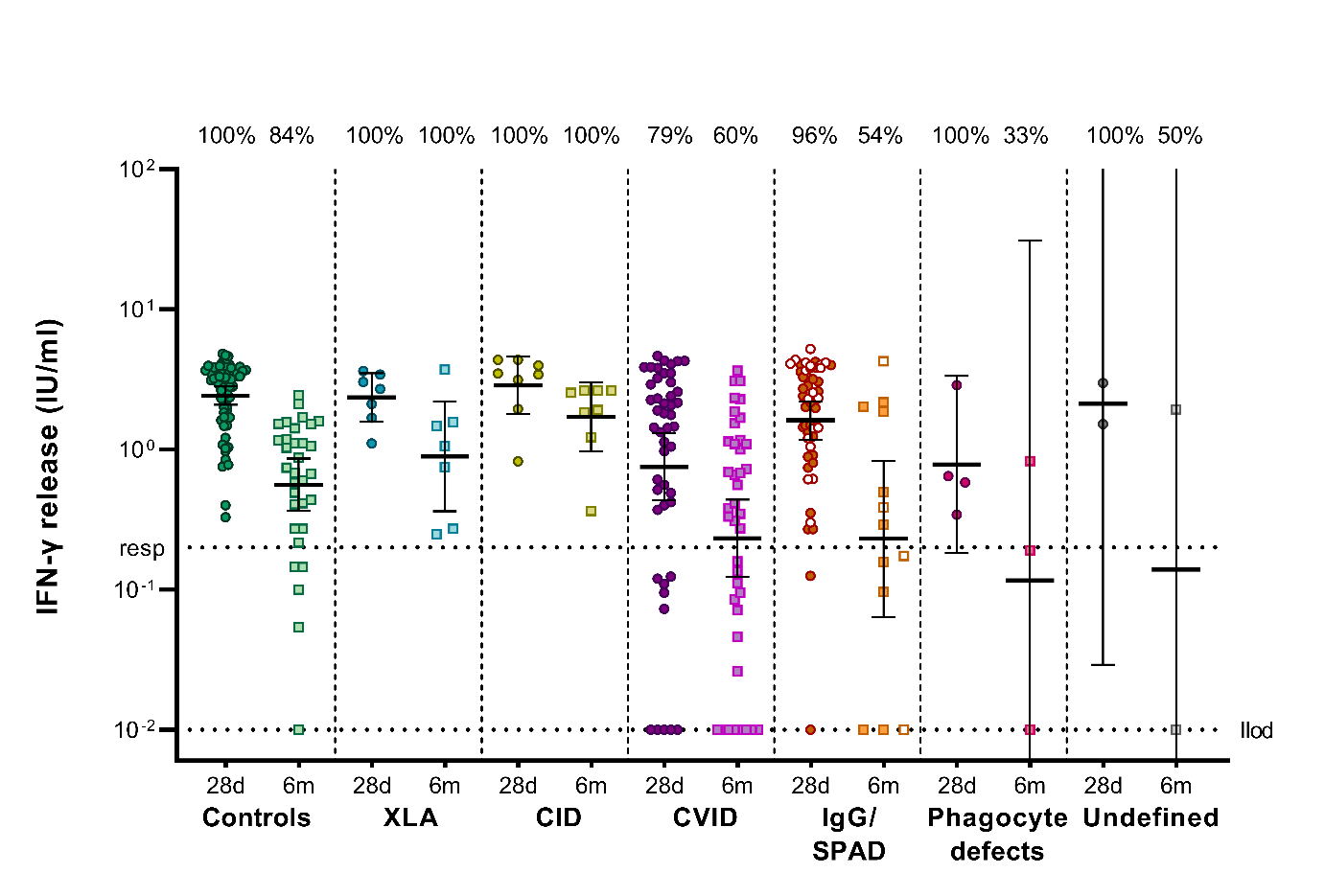 |
| --- |
| **Fig. 6 SARS-CoV-2-specific T-cell responses 28 days and six months after the second COVID-19 vaccination obtained with a EuroImmun assay.**SARS-CoV-2-specific T-cell responses measured by an IFN-γ release assay (EuroImmun) after stimulation with a receptor binding domain peptide pool of whole blood obtained 28 days and six months after the second vaccination. Lower limit of detection (llod) is 0.01 IU/ml and responder cut off (resp) was 0.2 IU/ml. The percentage of responders is indicated above the graph. Results are expressed as international units/milliliter (IU/mL). Line indicates the geometric mean, error bars indicate the 95% confidence interval. Within each cohort, IFN-γ levels at 28 days and six months were compared using Wilcoxon paired signed rank test. The SPAD cohort is indicated with white symbols while the IgG cohort is indicated with orange symbols. XLA = X-linked agammaglobulinemia, CID = Combined Immunodeficiency, CVID = Common Variable Immunodeficiency, IgG = Isolated IgG subclass deficiency ± IgA deficiency, SPAD = Specific polysaccharide antibody deficiency, Undefined = Undefined antibody deficiency. |

**Online Resource 10: Supplementary fig. 7**

| 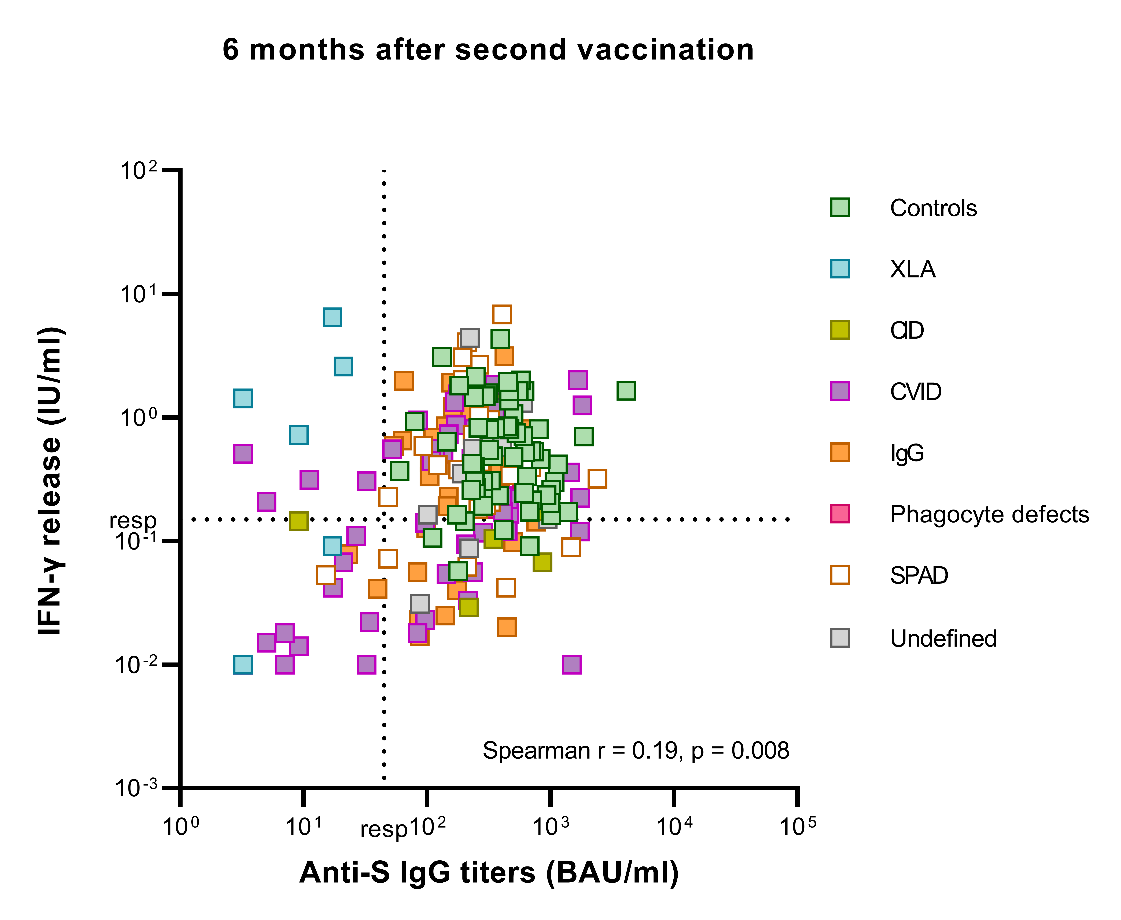 |
| --- |
| **Fig. 7 Correlation between IgG titers and T-cell responses six months after second COVID-19 vaccination**  The dotted horizontal line is the responder cut-off of the QIAGEN interferon-gamma release assay (0.15 IU/mL). The dotted vertical line is the responder cut-off of the Luminex assay (44.8 BAU/ml). Spearman’s ρ test was used to perform correlation analysis. XLA = X-linked agammaglobulinemia, CID = Combined Immunodeficiency, CVID = Common Variable Immunodeficiency, IgG = Isolated IgG subclass deficiency ± IgA deficiency, SPAD = Specific polysaccharide antibody deficiency, Undefined = Undefined antibody deficiency. |
